# Supplementary material for: A Stress-Responsive NAC Transcription Factor from Tiger Lily (LlNAC2) Interacts with LlDREB1 and LlZHFD4 and Enhances Various Abiotic Stress Tolerance in Arabidopsis
Source: Int J Mol Sci. 2019 Jun 30;20(13):3225. doi: 10.3390/ijms20133225 (PMC6651202; doi:10.3390/ijms20133225)

LlZHFD CDS translation

1 ATGGATCTCCCCATTTATCAAGGGGATATCCATGACAACCACAAC
M D L P I Y Q G D I H D N H N
46 AAAAGTGGGGCGGCGGCGGCGGTGAAGTACAAGGAGTGCCTGAAG
K S G A A A A V K Y K E C L K
91 AACCATGCAGCCACCATTGGAGTCATTGCCATTGATGGGTGTGGG
N H A A T I G V I A I D G C G
136 GAGTTCATGCCGAGTGGGGAGGAGGGCACACTGGAGGCCCTTAGA
E F M P S G E E G T L E A L R
181 TGCTCAGCCTGCGGCTGCCACCGGAACTTCCACAGAAAAGAGACT
C S A C G C H R N F H R K E T
226 GAAGGAGAGGCTTCATCGTGTGGGTGCTCCCACAAGCTCTTGTCA
E G E A S S C G C S H K L L S
271 CCGCACCATATGATCATGCCCTTGGCCCTGCAGGCCTCGGAGCCA
P H H M I M P L A L Q A S E P
316 TTTGGCATGGTGAGGAAGAGATTCAGGACCAAGTTCACGCCGGGG
F G M V R K R F R T K F T P G
361 CAGAAGGAGAAGATGCTAGGGTTTGCTGAAAGAGTTGGATGGAAG
Q K E K M L G F A E R V G W K
406 ATGCAGAGGCTGGATGAGGGTGTGGTGCAGCAGTTCTGTCAGGAG
M Q R L D E G V V Q Q F C Q E
451 GTTGGGGTGAAGAGGAGGGTGCTCAAGGTTTGGATGCACAACAAC
V G V K R R V L K V W M H N N
496 AAGCAGAATTTGGCAAAAAAGGATCCATTGCAGAGTGACTACTGA
K Q N L A K K D P L Q S D Y *

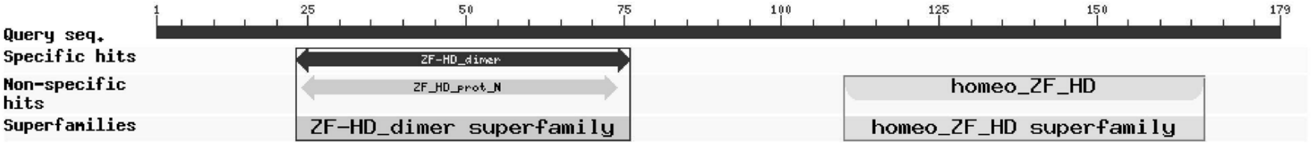

Supplement: Supplementary file 1 [file ijms-20-03225-s001.zip › Supplementary material/FigureS2.pdf]
