# Supplementary material for: A Stress-Responsive NAC Transcription Factor from Tiger Lily (LlNAC2) Interacts with LlDREB1 and LlZHFD4 and Enhances Various Abiotic Stress Tolerance in Arabidopsis
Source: Int J Mol Sci. 2019 Jun 30;20(13):3225. doi: 10.3390/ijms20133225 (PMC6651202; doi:10.3390/ijms20133225)
Supplement: Supplementary file 1 [file ijms-20-03225-s001.zip › Supplementary material/TableS1.docx]

**Supplementary Table S1 Primers used in this study.**

| **Primers name** | **Accession** | **Primer sequence (5’- 3’)** |
| --- | --- | --- |
| qRT-PCR *LlNAC2* |  | F: CGGAGGTCGGATTGA |
|  |  | R: TGGATGATTGGGTCTTG |
| qRT-PCR *LlTIP1* |  | F: CGAAGCCAGAAACGGAGAAGAAT |
|  |  | R: CGAAGCCAGAAACGGAGAAGAAT |
| qRT-PCR *Atactin* | NM_112764 | F: CTCATGCCATCCTCCGTCTT |
|  |  | R: ACTTGCCCATCGGGTAATTC |
| qRT-PCR *AtRD29A* | NM_124610 | F: GAGCAACGAGGGGAAGATAAAAG |
|  |  | R: TCAGTCGCACCACCACCGAACCA |
| qRT-PCR *AtRD29B* | NM_124609 | F: CAAAACCAAGCACCTACACA |
|  |  | R: CTCCTTCACTCCACTTCCAC |
| qRT-PCR *AtRD20* | NM_128898 | F: ATTCGAGCACCTATGACACC |
|  |  | R: AAACTTCCATCAAAGCAACC |
| qRT-PCR *AtCOR47* | NM_101894 | F: TCCCAGGACACCACGACAAGAC |
|  |  | R: CCTCTTCAGTGGTCTTGGCATG |
| qRT-PCR *AtGSTF6* | NM_100174 | F: ACTCTTCGACGAGCGTCCACAT |
|  |  | R: GGTCATCGCCACTTTTATTACA |
| qRT-PCR *At**APX2* | At3G09640 | F: TCAGGATTCGAGGGTGCATG |
|  |  | R: AAGGCATCCTCATCTGCAGC |
| qRT-PCR *At**LEA14* | At1G01470 | F: ACCGGATTTAATTCATTAAGCGCT |
|  |  | R: TCCCAAGCTGGCAGAGGGAAT |
| qRT-PCR *AtGolS1* | At2g47180 | F: AGCCGTTCATCACCGCTCTTAC |
|  |  | R: ACTCCTGGCAACATTCAAGCAG |
| qRT-PCR *GUS* |  | F: GGGCAACAAGCCGAAAGA |
|  |  | R: GCCAGTGGCGCGAAATAT |
| *LlNAC2* 5’-RACE |  | R1: GTCACCGCCAAACTGCATCATCCC |
|  |  | R2: ACGTGACCGGAGCAGCTCGAATCT |
| *LlNAC2* CDS |  | F: ATGGGCGGTCCAGATCTTC |
|  |  | R: CTAGAACGGCTTCAGCAAG |
| *LlNAC2* Promoter |  | GSP1: GTAGGCTGTTCTTGCGACGAGCGGATC |
|  |  | GSP2: GTACGTCACAAGCTCTTCGTCCGTTGGA |
| pBI121- LlNAC2-GFP |  | F: CATTTACGAACGATACTCGAG(XhoI)ATGGGCGGTCCAGATCTTC |
|  |  | R: CACCATCACTAGTACGTCGAC(SalI)GAACGGCTTCAGCAAGTGC |
| pGBKT7-LlNAC2 |  | F: CATGGAGGCCGAATTC(EcoRI)ATGGGCGGTCCAGATCTTC |
|  |  | R: GCAGGTCGACGGATCC(BamHI)CTAGAACGGCTTCAGCAAG |
| pGBKT7-LlNAC2-N |  | F: GCAGGTCGACGGATCC(BamHI)ATCCAGCCGTAGGCTGTTC |
|  |  | R: CATGGAGGCCGAATTC(EcoRI)GATGATTGGGTCTTGTGCC |
| pGADT7-LlDREB1 |  | F: GCCATGGAGGCCAGTGAATTC(EcoRI)CTCAGGATGTATACACCATC |
|  |  | R: CAGCTCGAGCTCGATGGATCC(BamHI)TCAATTCGACGACAGAATTGC |
| pAbAi-NAC-CRT/DRE |  | F: TGAATTCGAGACTCGGTACC(KpnI)AAACAATGACAGCTG |
|  |  | R: GGGGATCTGTCGACCTCGAG(XhoI)GTCGGCTGCGGATGT |
| YCE(M) |  | F: CGTTCCAACCACGTCTTCAAAG |
| YCE(M) |  | R: GTACAGCTCGTCCATGCC |
| YNE173 |  | F: CAATCCCACTATCCTTCGCAAG |
| YNE173 |  | R: GCTGAACTTGTGGCCGTTTAC |
