# Supplementary material for: A Stress-Responsive NAC Transcription Factor from Tiger Lily (LlNAC2) Interacts with LlDREB1 and LlZHFD4 and Enhances Various Abiotic Stress Tolerance in Arabidopsis
Source: Int J Mol Sci. 2019 Jun 30;20(13):3225. doi: 10.3390/ijms20133225 (PMC6651202; doi:10.3390/ijms20133225)
Supplement: Supplementary file 1 [file ijms-20-03225-s001.zip › Supplementary material/TableS2.docx]

**Supplementary Table S2 NCBI accession numbers of genes used in multiple sequence alignments and phylogenetic tree analysis**

| **Gene Name** | **NCBI Accession Number** |
| --- | --- |
| ZjNAC2 | XP_015885739.1 |
| MnNAC2 | XP_010095416.1 |
| EgNAC48 | XP_010930725.1 |
| ANAC002 | [NM_100054.3](https://www.ncbi.nlm.nih.gov/nuccore/NM_100054.3) |
| GmNAC2 | NP_001236871.2 |
| ONAC048 | [XM_015765434.2](https://www.ncbi.nlm.nih.gov/nuccore/XM_015765434.2) |
| ANAC002/ATAF1 | [NM_100054.3](https://www.ncbi.nlm.nih.gov/nuccore/NM_100054.3) |
| ONAC071 | [XM_015761800.2](https://www.ncbi.nlm.nih.gov/nuccore/XM_015761800.2) |
| ANAC081/ATAF2 | [NM_147856.4](https://www.ncbi.nlm.nih.gov/nuccore/NM_147856.4) |
| ANAC102 | NP_001330407.1 |
| ONAC002 | [XM_015775072.2](https://www.ncbi.nlm.nih.gov/nuccore/XM_015775072.2) |
| ONAC068 | [XM_015768220.2](https://www.ncbi.nlm.nih.gov/nuccore/XM_015768220.2) |
| ANAC072 | [NM_118875.4](https://www.ncbi.nlm.nih.gov/nuccore/NM_118875.4) |
| ANAC019 | NP_175697.1 |
| ANAC047 | NP_001118568.1 |
| ANAC029 | NP_564966.1 |
| ANAC025 | NP_564771.1 |
| ANAC056 | NP_188170.1 |
| ANAC092 | NP_198777.1 |
| ONAC045 | [XM_015760382.2](https://www.ncbi.nlm.nih.gov/nuccore/XM_015760382.2) |
| ONAC022 | [NM_127259.4](https://www.ncbi.nlm.nih.gov/nuccore/NM_127259.4) |
| ANAC016 | NP_001322918.1 |
