# Supplementary material for: A Stress-Responsive NAC Transcription Factor from Tiger Lily (LlNAC2) Interacts with LlDREB1 and LlZHFD4 and Enhances Various Abiotic Stress Tolerance in Arabidopsis
Source: Int J Mol Sci. 2019 Jun 30;20(13):3225. doi: 10.3390/ijms20133225 (PMC6651202; doi:10.3390/ijms20133225)
Supplement: Supplementary file 1 [file ijms-20-03225-s001.zip › Supplementary material/TableS3.docx]

**Supplementary Table S3 Stress-related cis-acting regulatory elements identiﬁed in the promoter region of *LlNAC2***

| **Site name** | **(Strand) Position** | **Sequence** | **Function** |
| --- | --- | --- | --- |
| ARE | ( + ) 78, 429;  (－) 133, 581, 634 | TGGTTT | cis-acting regulatory element essential for the anaerobic induction |
| CRT/DRE | ( + ) 312, 351;  (－) 487 | G(C/T)CGAC | Core CRT/DRE motif |
| LTRE | ( + ) 312 | CCGAC | Putative low temperature responsive element |
| MYBRS | ( + ) 548, 583;  (－) 1279, 1442 | (A/T)AACCA | MYB recognition site involved in drought inducibility and ABA signaling |
| MYCRS | ( + ) 487, 1014, 1064, 1070, 1091, 1170, 1191, 1281 | CA(A/G)CTG, CAT(A/T/C/G)TG, CAC(A/G)TG | MYC recognition site involved in cold and drought-inducibility |
| [CGTCA-motif](http://bioinformatics.psb.ugent.be/webtools/plantcare/cgi-bin/show_site_info.htpl?QWhere=ID_of_Site%20like%20'HV~CGTCA-motif'&StartAt=0&NbRecs=10) | ( + )172;  (－) 277, 471 | CGTCA | cis-acting regulatory element involved in the MeJA-responsiveness |
| ABRE | ( + ) 162, 211 | CACGTGGC | cis-acting element involved in the ABA responsiveness |
| DPBF | ( + ) 680, 1245;  (－) 670, 1067, 1086, 1198, 1301 | ACAC(A/C)CG, ACACG(A/T)G | [DPBF1&2 binding site motif](http://bioinfo.cau.edu.cn/ProFITS/BS_anno.php?source=PLACE&BS=DPBFCOREDCDC3) induced by ABA |
| ARF | ( + ) 1118 | TGTCTC | ARF (auxin response factor) binding site |
| [TGA-element](http://bioinformatics.psb.ugent.be/webtools/plantcare/cgi-bin/show_site_info.htpl?QWhere=ID_of_Site%20like%20'BO~TGA-element'&StartAt=0&NbRecs=10) | ( + ) 743, 1027 | AACGAC | auxin-responsive element |
