# Supplementary figures and images for: A Stress-Responsive NAC Transcription Factor from Tiger Lily (LlNAC2) Interacts with LlDREB1 and LlZHFD4 and Enhances Various Abiotic Stress Tolerance in Arabidopsis
Source: Int J Mol Sci. 2019 Jun 30;20(13):3225. doi: 10.3390/ijms20133225 (PMC6651202; doi:10.3390/ijms20133225)

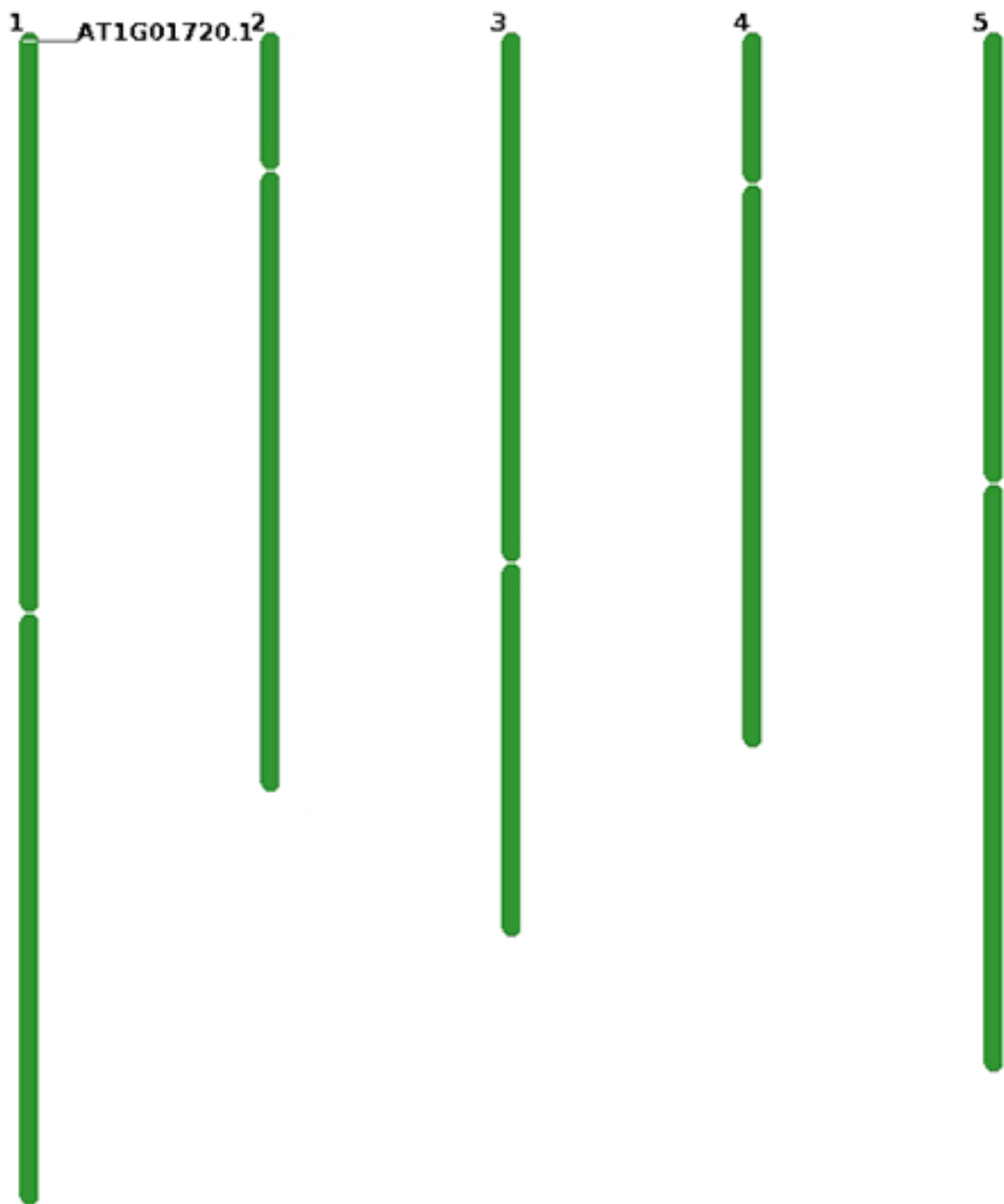

Supplement: Supplementary file 1 [file ijms-20-03225-s001.zip › Supplementary material/FigureS1.pdf]

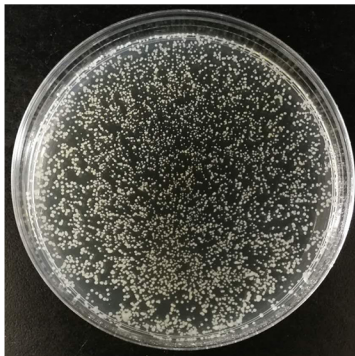

AbA 0 ng/ml

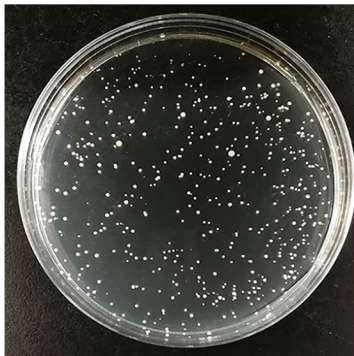

100ng/ml

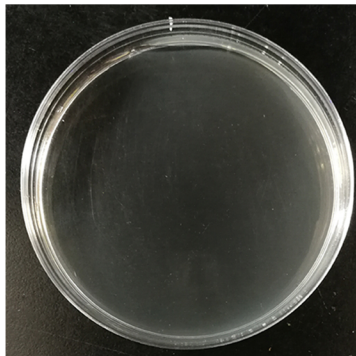

150ng/ml

Supplement: Supplementary file 1 [file ijms-20-03225-s001.zip › Supplementary material/FigureS3.pdf]

Marker

1

2

3

4

5

6

7

8

9

10

11

12

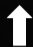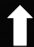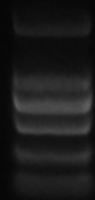

Supplement: Supplementary file 1 [file ijms-20-03225-s001.zip › Supplementary material/FigureS4.pdf]
